# The synthetic antimicrobial peptide 19-2.5 attenuates septic cardiomyopathy and prevents down-regulation of SERCA2 in polymicrobial sepsis

**DOI:** 10.1038/srep37277

**Published:** 2016-11-17

**Authors:** Lukas Martin, Klemens Horst, Fausto Chiazza, Silvia Oggero, Massimo Collino, Klaus Brandenburg, Frank Hildebrand, Gernot Marx, Christoph Thiemermann, Tobias Schuerholz

**Affiliations:** 1 Department of Intensive Care and Intermediate Care, University Hospital RWTH Aachen, Aachen, Germany; 2 The William Harvey Research Institute, Barts & The London School of Medicine & Dentistry, Queen Mary University of London, London, UK; 3 Department of Orthopaedic Trauma, University Hospital RWTH Aachen, Aachen, Germany; 4 Department of Drug Science & Technology, University of Turin, Turin, Italy; 5 Division of Biophysics, Forschungszentrum Borstel, Borstel, Germany

## Abstract

An impairment of cardiac function is a key feature of the cardiovascular failure associated with sepsis. Although there is some evidence that suppression of sarcoplasmic reticulum Ca^2+^-ATP-ase (SERCA2) contributes to septic cardiomyopathy, it is not known whether prevention of the down-regulation of SERCA2 improves outcome in sepsis. Thus, we investigated whether the administration of the synthetic antimicrobial peptide Pep2.5 may attenuate the cardiac dysfunction in murine polymicrobial sepsis through regulating SERCA2 expression. We show here for the first time that the infusion of Pep2.5 reduces the impaired systolic and diastolic contractility and improves the survival time in polymicrobial sepsis. Preservation of cardiac function in sepsis by Pep2.5 is associated with prevention of the activation of NF-κB and activation of the Akt/eNOS survival pathways. Most notably, Pep2.5 prevented the down-regulation of SERCA2 expression in a) murine heart samples obtained from mice with sepsis and b) in cardiomyocytes exposed to serum from septic shock patients. Thus, we speculate that Pep2.5 may be able to prevent down-regulation of cardiac SERCA2 expression in patients with sepsis, which, in turn, may improve cardiac function and outcome in these patients.

Sepsis is defined as a life-threatening organ dysfunction caused by a dysregulated host response to infection, with the heart as one of the most frequently affected organs^1^. The presence of septic cardiomyopathy indicates a worse prognosis with mortality rates of up to 70%^2^. In particular, septic cardiomyopathy develops as the result of myocardial calcium (Ca^2+^) dysregulation^3^. While Ca^2+^ entry is determined solely by the quantity of membrane L-type Ca^2+^ channels (LTCC), the amount of Ca^2+^ stored in the sarcoplasmic reticulum (SR) and available for cytosolic release is mainly regulated through the SR Ca^2+^-ATP-ase (SERCA2)^3^. The inhibition of SERCA2 leads to a fail in diastolic relaxation, secondary to the blocked reuptake of Ca^2+^ into the SR^4^. Although limited evidence indicates that the function of SERCA2 maybe impaired or downregulated in lipopolysaccharide (LPS)-challenged mice, it is unknown whether murine CLP-sepsis affects the expression of SERCA^5^. Furthermore, it is not known whether prevention of the down-regulation of SERCA2 improves cardiac function in sepsis.

The majority of cases of septic cardiomyopathy is caused by a disproportional immune response to pathogen associated molecular patterns (PAMPs), i.e. LPS from Gram-negative and lipoproteins/-peptides (LP) from Gram-positive bacteria^6^. Conventional antibiotics may kill bacteria, but at the same time, release bacteria-derived wall-fragments, such as LPS or LP, which in turn cause systemic and cardiac inflammation and Ca^2+^-dysregulation during septic cardiomyopathy^7^.

Antimicrobial peptides are known to kill bacteria without releasing pro-inflammatory factors, but translation of preclinical findings to patients with sepsis was limited by high cell toxicity^7^. The newly designed *synthetic* antimicrobial peptide 19-2.5 (Pep2.5) belongs to the class of synthetic anti-lipopolysaccharide peptides (SALP = synthetic anti-LPS peptides). However, its activity is not restricted to Gram-negative bacterial infection^8^, as Pep2.5 neutralizes LPS as well as lipoteichoic acid (LTA) and LPs without causing harm^9^.

The present study was designed to evaluate the effects of Pep2.5 in (a) a murine model of polymicrobial sepsis using cecal-ligation and puncture (CLP) to induce septic cardiomyopathy and (b) an *in vitro* model of cardiomyocytes exposed to human sepsis serum (for a translational approach). Having discovered that Pep2.5 attenuates the cardiomyopathy caused by sepsis, we have then investigated the effects of Pep2.5 on SERCA2 expression.

## Methods

Additional details relating to materials and methodology are provided in the *Supplementary*.

### Use of human subjects-ethic statement

All patients or their legal representative gave written informed consent. Before inclusion of the first individual, the local ethics committee (University Hospital RWTH Aachen, EK 206_09) approved this study, which was performed in accordance with the Declaration of Helsinki in its latest form.

### Human materials

Human serum samples were collected from a cohort of patients admitted to the intensive care unit of the University Hospital RWTH Aachen^10^. All samples are stored in the centralized Biomaterial Database (RWTH cBMB) of the University Hospital RWTH Aachen. All samples (n = 10) were prospectively collected within 24 h after the patients fulfilled criteria of septic shock, according to the 3^rd^ international consensus definitions (sepsis-3)^1^. Further information on patients characteristics are displayed in Supplementary Table S1.

### Use of experimental animals-ethic statement

All animal experiments have been performed in accordance with the guidelines of the Institutional Animal Care and Use Committee (IACUC) and the National Animal Welfare Law after approval by the responsible government authority (LANUV-NRW, Germany: AZ 84–02.04.2014.4300).

### Murine sepsis model

This study was carried out on 2-month-old male Naval Medical Research Institute (NMRI)-mice weighing 32 ± 3 g, receiving a standard diet and tap water *ad libitum*. The murine sepsis model was divided into three steps^11^,^12^: In a first step, a central vein catheter (PE-tube, self-made) was implanted in the jugular vein. Mice were transferred back into the cage to rest for 48 h. The i.v.-line was connected to the syringe pump, and animals received saline (of 100 μl/h) as fluid resuscitation. In a second step, mice underwent CLP (18-G needle, double puncture) or sham operation^11^,^12^. Sham mice were not subjected to CLP, but were otherwise treated in the same way. All mice received a fluid resuscitation (0.9% saline; 200 μl *i.v.*) and analgesic therapy (buprenorphine; 0.05 mg/kg *s.c.*). Mice were treated with Pep2.5 (2.0 μg/h in saline 0.9%; 100 μl/h *i.v.*) or vehicle (0.9% saline; 100 μl/h *i.v.*). A summary of the experimental setup is provided in Supplementary Fig. S2.

### Quantification of cardiac dysfunction

Cardiac function was assessed in mice 24 h subsequent to CLP, *via* 1.4-F pressure volume catheter (SPR 839, Millar Instruments, Houston, Texas, USA)^13^. Then, the experiment was terminated and organ and blood samples were collected for quantification of cardiac dysfunction and injury. Further information on quantification of cardiac dysfunction are mentioned in the online *Supplementary*.

### Peptide-synthesis

The synthesis and purification of Pep2.5 was performed as described before^8^,^12^. The used batches were produced by BACHEM (Bubendorf, Switzerland). The amino acid sequence of this 20’mer is GCKKYRRFRWKFKGKFWFWG, with a molecular weight of 2711 kDa. Pep2.5 was amidated at the C-terminal end and had a purity of >95% as measured by HPLC and MALDI-TOF mass spectrometry^9^.

### Cardiomyocyte cell culture

HL-1 cardiomyocytes, originally gifted from William Claycomb (Louisiana State University, Baton Rouge, Louisiana, USA) were cultured in Claycomb medium as described previously^14^,^15^. Briefly, HL-1 cells were grown on 5 mg/ml fibronectin and 0.02% gelatine. HL-1 cells were maintained in supplemented Claycomb medium and incubated under an atmosphere of 5% CO_2_ and 95% air at 37 °C. Cells were passaged by adding trypsin-EDTA to the culture dishes for 5–10 min. Trypsin activity was blocked by its inhibitor glycine max at a ratio of 10 ml per 1 cm^2^ of cells. As described before^14^,^15^, HL-1 cells were plated on six-well plates and exposed to serum of septic shock patients (5% in supplemented Claycomb medium) in the presence or absence of peptide 19-2.5 (20 μg/ml) for 24 h.

### Immunofluorescence

Immunofluorescence assays were carried out as described before^16^,^17^. The monoclonal mouse anti-SERCA2 antibody (MA3-919, dilution 1:200; Thermo Fischer, Rockford, Illinois, USA) has been used for visualization of SERCA2 in murine heart tissue and HL-1 cells. Microscopy was performed with an Axiostar/MRc5 and LSM 710 (Zeiss, Jena, Germany). Images were acquired with AxioVision (Zeiss, Jena, Germany) and intensity profiles were quantified with ImageJ 1.48 (NIH, Bethesda, Maryland, USA).

### Immunoblot analysis

Semi-quantitative immunoblot analyses of nuclear translocation of p65 and the phosphorylation of IκBα, IKKα/β, Akt, and eNOS were carried out in mouse heart tissues as described previously^18^. Immunoblot analyses of SERCA2 in mouse heart tissues and HL-1 cells have been performed as described before^17^, using the monoclonal mouse anti-SERCA2 antibody (MA3-919, dilution 1:200; Thermo Fischer, Rockford, Illinois, USA).

### RNA extraction and PCR

Extraction of total RNA and PCR were performed as described previously^14^. For quantitative real-time PCR the following primers were used: SERCA 2 5′ *CCATCTGCTTGTCCATGTCAC* 3′ (for) and 5′ *CAAATGGTTTAGGAAGCGGTTACT* 3′ (rev). Ribosomal Protein S7 was used as an endogenous normalization control: 5′ *GGTGGTCGGAAAGCTATCA* 3′ (for) and 5′ *AAGTCCTCAAGGATGGCGT* 3′ (rev).

### Statistics

Unless otherwise stated, data are presented as mean ± standard deviation (SD) of *n* observations, where *n* represents the number of animals/experiments studied. Due to relatively low n-numbers, data were not regarded as normally distributed. Therefore, we assessed data by Kruskal-Wallis test and Dunn’s test (corrected for multiple comparisons) using SPSS Statistics 20.0 for Windows (SPSS Inc. Chicago, Illinois, USA) and GraphPad Prism 6 (GraphPad, San Diego, California, USA). A P-value of less than 0.05 was considered to be statistically significant.

## Results

Additional results are provided in the online *Supplementary*.

### Impairment of baseline haemodynamic following polymicrobial sepsis is attenuated by Pep2.5 treatment

**Figure 1:**
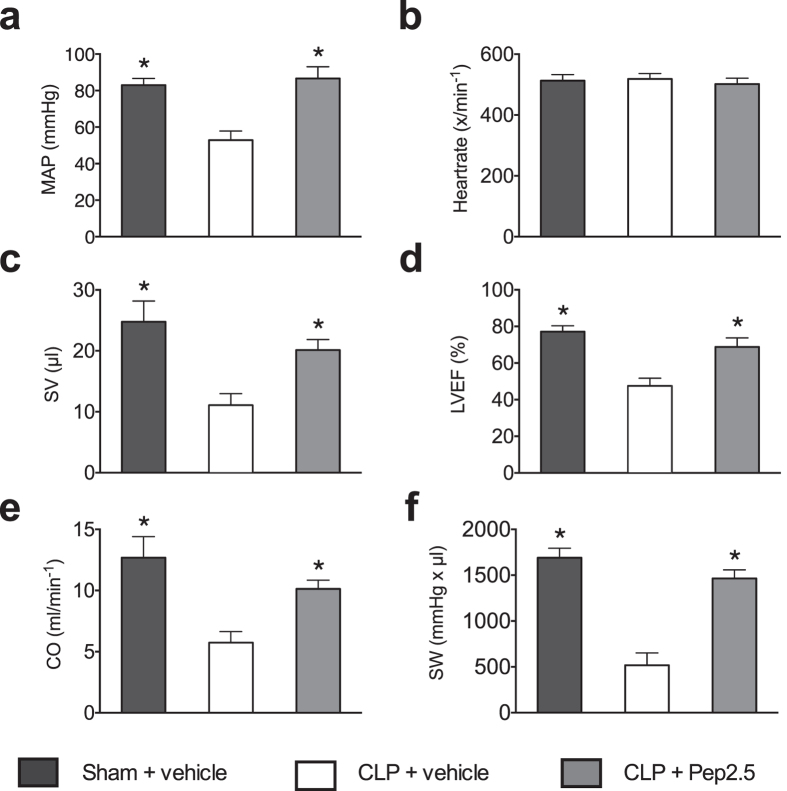
Effect of cecal ligation and puncture and treatment with Pep2.5 on baseline haemodynamic. (**a**) Mean arterial pressure (MAP), (**b**) heart rate, (**c**) stroke volume (SV), (**d**) left ventricular ejection fraction (LVEF), (**e**) cardiac output (CO), and (**f**) stroke work (SW) were assessed by pressure volume catheter 24 h subsequent to sham or CLP in 2-month-old male NMRI-mice. After CLP mice were treated with Pep2.5 (2.0 μg/h in saline 0.9%) or vehicle (100 μl/h saline 0.9%). The following groups were studied: sham + vehicle (n = 8); CLP + vehicle (n = 8); CLP + Pep2.5 (n = 8). Data are expressed as means ± SD for *n* number of observations. *P < 0.05 vs. CLP + vehicle (Kruskall-Wallis test with Dunn’s multiple comparisons test).

The synthetic antimicrobial Pep2.5 limits systemic inflammation in CLP-challenged mice without causing harm^12^. Intravenous administration of Pep2.5 significantly attenuated the inflammatory response in CLP-challenged mice, indicated by significant lower plasma levels of the cytokines interleukin-6 (*P* = 0.0198), interleukin-10 (*P* = 0.0054), monocyte chemoattractant protein-1 (*P* = 0.0005), C-X-C motif ligand 1 (*P* = 0.0001), and interleukin-1β (*P* < 0.0001), respectively (Supplementary Fig. S3). We then investigated the effect of Pep2.5 on baseline (after insertion of catheter, but before any preload alterations) haemodynamic following polymicrobial sepsis in mice. Baseline global hemodynamic data were obtained 24 h subsequent to CLP-challenge *via* insertion of a 1.4-F conductance catheter in the right carotid and afterwards in the left ventricle. When compared to the sham animals, mice subjected to CLP demonstrated a significant decrease in mean arterial pressure (MAP; *P* = 0.0094), stroke volume (SV; *P* = 0.0001), left ventricular ejection fraction (LVEF; *P* < 0.0001), cardiac output (CO; *P* < 0.0002), and stroke work (SW; *P* < 0.0001), indicating an impairment of global haemodynamics (Fig. 1a,c–f). There was no difference in heart rate between CLP-challenged and sham animals (Fig. 1b). Intravenous administration of Pep2.5 (2.0 μg/h in 0.9% saline, 100 μl/h) significantly attenuated the impairment in baseline haemodynamics caused by sepsis, as confirmed by significantly higher values of MAP (*P* = 0.0004), SV (*P* = 0.0216), LVEF (*P* = 0.0296), CO (*P* = 0.0140), and SW (*P* = 0.0327), respectively (Fig. 1a,c–f).

### Cardiac dysfunction following polymicrobial sepsis is attenuated by Pep2.5 treatment

**Figure 2:**
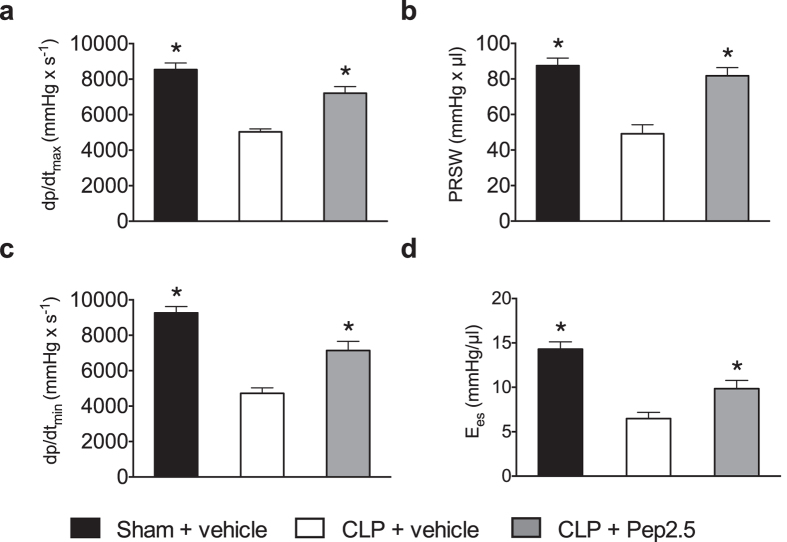
Effect of cecal ligation and puncture and treatment with Pep2.5 on myocardial contractility and prelaod related haemodynamic. (**a**) Maximal developed pressure (dp/dt_max_), (**b**) preload recruited stroke work (PRSW), (**c**) minimal developed pressure (dp/dt_min_), and (**d**) changes in left ventricular elastance (E_es_) were assessed by pressure volume catheter 24 h subsequent to sham or CLP in 2-month-old male NMRI-mice. After CLP mice were treated with Pep2.5 (2.0 μg/h in saline 0.9%) or vehicle (100 μl/h saline 0.9%). The following groups were studied: sham + vehicle (n = 8); CLP + vehicle (n = 8); CLP + Pep2.5 (n = 8). Data are expressed as means ± SD for *n* number of observations. *P < 0.05 vs. CLP + vehicle (Kruskall-Wallis test with Dunn’s multiple comparisons test).

Initially, myocardial contractility was measured in the steady state using the first derivative of developed pressure (dp/dt). When compared to the sham animals, mice subjected to CLP demonstrated a significant decrease in dp/dt_max_ (*P* = 0.0001), and dp/dt_min_ (*P* < 0.0001), respectively, indicating a strongly impaired systolic and diastolic contractility (Fig. 2a,c). These alterations were significantly attenuated in septic animals treated with Pep2.5. Intravenous administration of Pep2.5 (2.0 μg/h in 0.9% saline, 100 μl/h) significantly improved dp/dt_max_ (*P* = 0.0240), and dp/dt_min_ (*P* < 0.0001), respectively, indicating a therapeutic effect of Pep2.5 on systolic and diastolic contractility (Fig. 2a,c). Next, we evaluated left ventricular contractility under altered preload conditions by transient occlusion of the inferior vena cava with a cotton tip applicator from the opened abdominal cavity^13^. This methodical approach enables the measurement of the left ventricular performance independently from loading conditions (volume status)^13^. When compared to the sham animals, mice subjected to CLP showed a significant decrease in preload recruited stroke work (PRSW; *P* = 0.0001), indicating the presence of an impairment in systolic contractility independent of either preload or afterload^19^ (Fig. 2b). To evaluate the end-systolic stiffness of the myocardium, which is the most useful value to assess acute changes of contractile function^13^,^20^, we determined end-systolic left ventricular elastance (E_es_). When compared to the sham animals, mice subjected to CLP showed a significant decrease in E_es_ (*P* < 0.0001), confirming an impaired systolic contractility (Fig. 2d). The intravenous administration of Pep2.5 (2.0 μg/h in 0.9% saline, 100 μl/h) significantly attenuated this impairment in systolic and diastolic contractility, as confirmed by significantly higher values of dp/dt_max_ (*P* < 0.0001), dp/dt_min_ (P < 0.0001), PRSW (*P* = 0.0240), and E_es_ (*P* = 0.0041), in septic animals treated with Pep2.5, respectively (Fig. 2a–d).

### Survival time following polymicrobial sepsis is improved by Pep2.5 treatment

**Figure 3:**
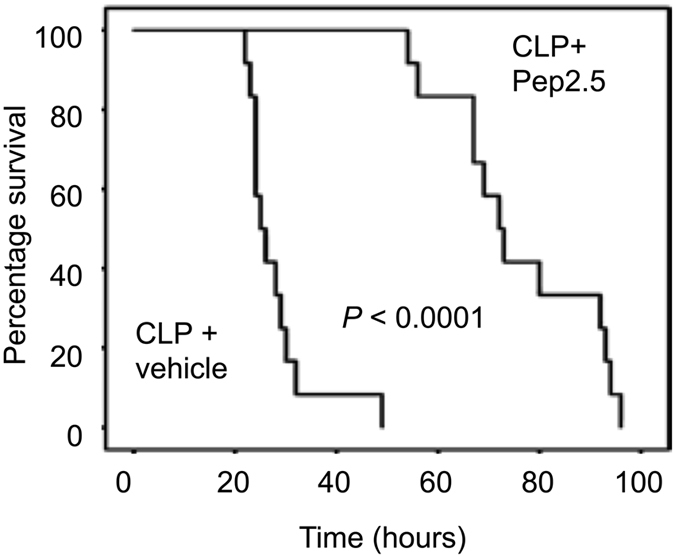
Effect of cecal ligation and puncture and treatment with Pep2.5 on survival. Survival rate of 2-month-old male NMRI-mice were assessed subsequent to CLP. After CLP mice were treated with Pep2.5 (2.0 μg/h in saline 0.9%) or vehicle (100 μl/h saline 0.9%). The following groups were studied: CLP + vehicle (n = 12); CLP + Pep2.5 (n = 12). Results were globally analysed by means of Kaplan-Meier survival analysis for *n* number of observations.

To get a better understanding of the importance of the reduction in cardiomyopathy afforded by Pep2.5 in sepsis for overall outcome, we investigated the effect of Pep2.5 on 100-h survival in mice with CLP. Mice subjected to CLP showed a progressive increase in mortality and all animals with CLP (that had been resuscitated with fluids and analgesics, but not Pep2.5) died within 46 hours. The intravenous administration of Pep2.5 (2.0 μg/h in 0.9% saline, 100 μl/h) significantly prolonged survival to 96 hours (*P* < 0.0001) (Fig. 3), but in the absence of antibiotic therapy was unable to prevent the death of animals from sepsis.

### Effect of polymicrobial sepsis and treatment with Pep2.5 on SERCA2 expression in mouse heart tissue

**Figure 4:**
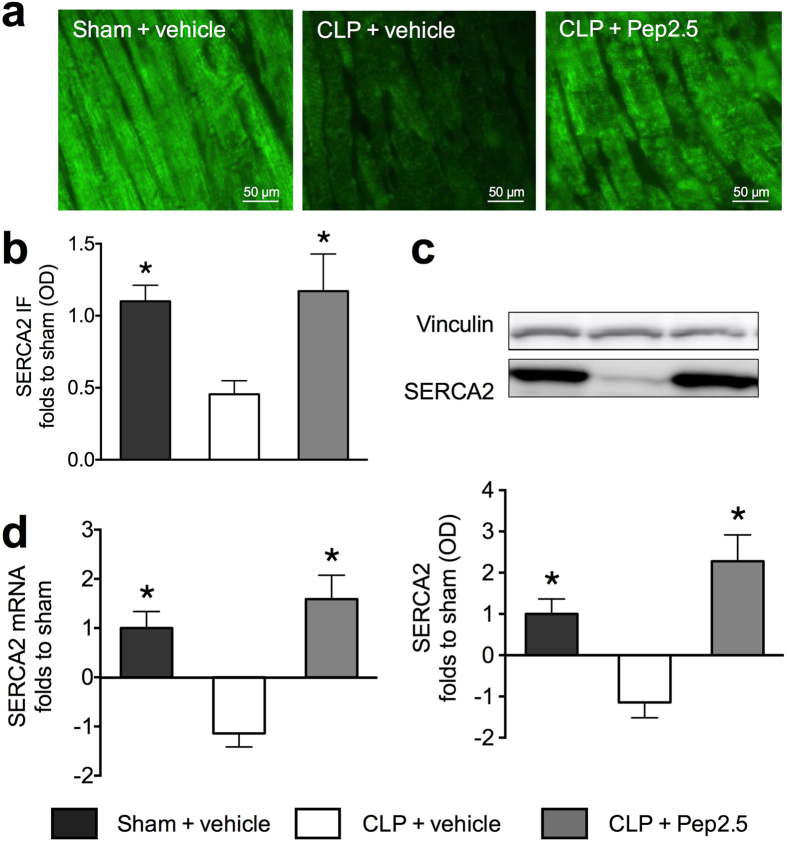
Effect of cecal ligation and puncture and treatment with Pep2.5 on SERCA2 expression in murine heart tissue. After CLP or sham 2-month-old male NMRI mice were treated with Pep2.5 (2.0 μg/h in saline 0.9%) or vehicle (100 μl/h saline 0.9%). (**a**) Representative immunofluorescence images of heart with antibodies reactive to sarcoplasmic reticulum Ca^2+^-ATP-ase (SERCA2) (green). (**b**) Quantification of SERCA2 expression in heart tissue was assessed 24 subsequent to sham or CLP by immunofluorescence and shown as relative optical density (OD); (**c**) Representative immunoblot of SERCA2 (bottom) and vinculin (top). OD of SERCA2 bands corrected for the corresponding vinculin bands and normalized using the related sham band; (**d**) Relative mRNA expression of SERCA2 corrected for S7 expression and normalized to sham. The following groups were studied: sham + vehicle (n = 8); CLP + vehicle (n = 8); CLP + Pep2.5 (n = 8). Data are expressed as means ± SD for *n* number of observations. *P < 0.05 vs. CLP + vehicle (Kruskall-Wallis test with Dunn’s multiple comparisons test).

**Figure 5:**
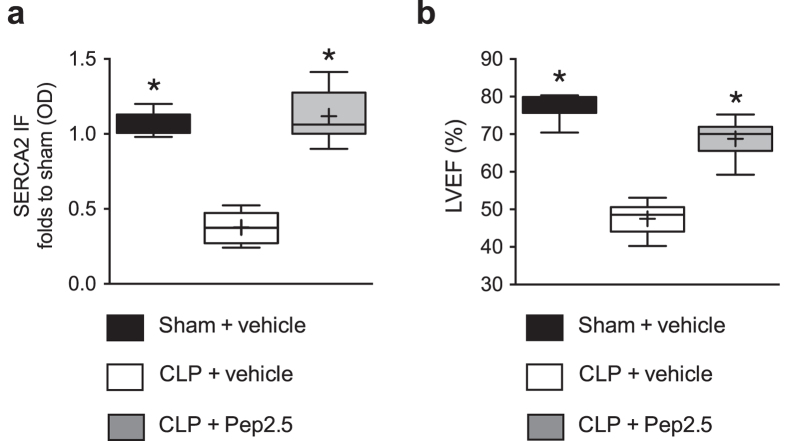
Effect of cecal ligation and puncture and treatment with Pep2.5 on SERCA2 expression in heart tissue and cardiac function in mice. After CLP or sham 2-month-old male NMRI mice were treated with Pep2.5. (**a**) Sarcoplasmic reticulum Ca^2+^-ATP-ase (SERCA2) expression was assessed by IF, or (**b**) the percentage ejection fraction (LVEF) was assessed by pressure volume catheter. The following groups were studied: sham + vehicle (n = 8); CLP + vehicle (n = 8); CLP + Pep2.5 (n = 8). Data are expressed as box and whiskers (min to max) for *n* number of observations.+ = means. *P < 0.0001 vs. CLP + vehicle (Kruskall-Wallis test with Dunn’s multiple comparisons test).

It has been reported that LPS-challenge in mice results in a decrease of SERCA2 expression in the heart (determined at 4 and 7 h, but not later) after administration of LPS, which contributes to cardiac dysfunction^21^. Thus, we investigated the effect of polymicrobial sepsis and treatment with Pep2.5 on SERCA2 expression in the mouse heart (Fig. 4). When compared to the sham animals, mice subjected to CLP showed a significant decrease of relative SERCA2 mRNA (*P* < 0.0001) and SERCA2 protein expression measured by western blot (*P* < 0.0001) and immunofluorescence (*P* = 0.0036) (Fig. 4). The intravenous administration of Pep2.5 (2.0 μg/h in 0.9% saline, 100 μl/h) significantly attenuated the decrease of SERCA2 mRNA (*P* < 0.0001) and SERCA2 protein expression measured by western blot (*P* < 0.0001) and immunofluorescence (*P* = 0.0026) in mouse heart tissue (Fig. 4). The observed reduction in SERCA2 expression was associated with a decline in LVEF and, hence, cardiac dysfunction. Most notably, prevention by Pep2.5 of the reduction in SERCA2 expression was also associated with an improvement in LVEF, strongly suggesting that prevention of SERCA2 expression by Pep2.5 contributes to or accounts for the improvement in cardiac function seen in septic mice treated with the peptide *in vivo* (Fig. 5).

### Effects of polymicrobial sepsis and treatment with Pep2.5 on the nuclear translocation of p65 and the phosphorylation of IKKα/β and IκBα in murine heart tissue

**Figure 6:**
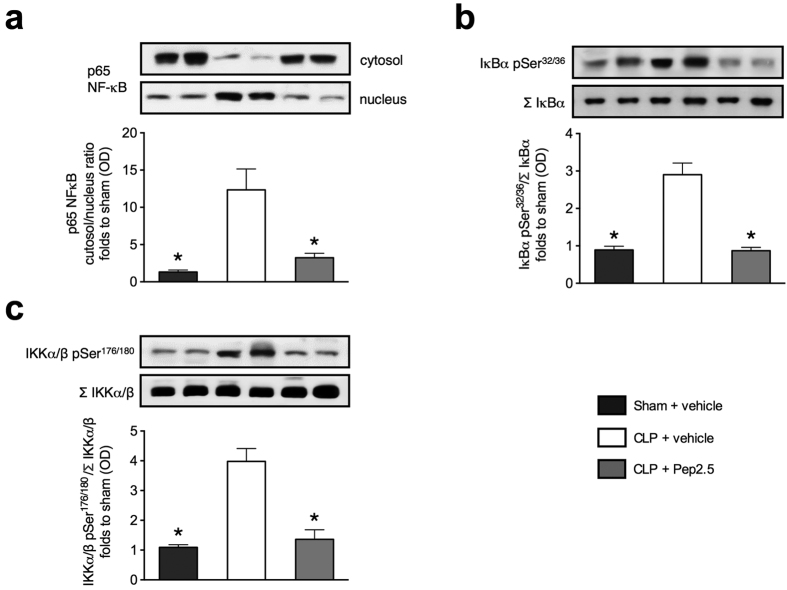
Effect of cecal ligation and puncture and treatment with Pep2.5 on the nuclear translocation of p65 and the phosphorylation of IκBα and IKKα/β in murine heart tissue. After CLP or sham 2-month-old male NMRI mice were treated with Pep2.5 (2.0 μg/h in saline 0.9%) or vehicle (100 μl/h saline 0.9%). Signaling events in heart tissue were assessed at 24 h subsequent to CLP. Each immunoblot is from a single experiment and is representative of five separate experiments. Densitometric analysis of the bands is expressed as relative optical density (OD) of (**a**) NFĸB p65 subunit levels in both cytosolic and nuclear fractions expressed as a nucleus:cytosol ratio; (**b**) phosphorylated IκBα (pSer^32/36^) corrected for the corresponding total IκBα content (Σ IκBα); (**c**) phosphorylated IKKα/β (pSer^176/180^) corrected for the corresponding total IKKα/β content (Σ IKKα/β). All values were corrected for the corresponding Tubulin and normalized using the related sham-operated band. The following groups were studied: sham + vehicle; CLP + vehicle; CLP + Pep2.5. Data are expressed as means ± SD for *n* number of observations. *P < 0.0001 vs. CLP + vehicle (Kruskall-Wallis test with Dunn’s multiple comparisons test).

To gain a better insight into the potential mechanism(s) underlying the observed therapeutic effects of Pep2.5 in septic cardiomyopathy, we investigated key signalling events in heart tissue 24 h subsequent to CLP. When compared with sham mice, CLP-challenged mice demonstrated a significant increase in the nuclear translocation of the p65 subunit of NF-κB (nuclear factor κ-light-chain-enhancer of activated B cells) (Fig. 6a) (P = 0.0026) as well as a significantly increased degree of phosphorylation of IκB kinas α and β (IKKα/β) on Ser^176/180^ (*P* = 0.0002) (Fig. 6b) and of IκBα on Ser^32/36^ (*P* < 0.0001) (Fig. 6c). The intravenous administration of Pep2.5 (2.0 μg/h in 0.9% saline, 100 μl/h) resulted in a significant attenuation of the nuclear translocation of the NF-κB subunit p65 (*P* = 0.0064) (Fig. 6a), the phosphorylation of IKKα/β on Ser^176/180^ (*P* = 0.0003) (Fig. 6b), and of IκBα on Ser^32/36^ (*P* < 0.0001) (Fig. 6c).

### Effects of polymicrobial sepsis and treatment with Pep2.5 on Akt and eNOS phosphorylation in murine heart tissue

**Figure 7:**
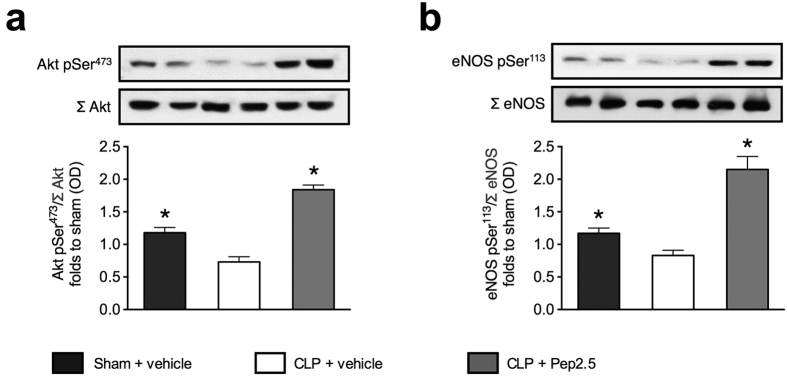
Effect of cecal ligation and puncture and treatment with Pep2.5 on Akt and eNOS phosphorylation in murine heart tissue. After CLP or sham 2-month-old male NMRI mice were treated with Pep2.5 (2.0 μg/h in saline 0.9%) or vehicle (100 μl/h saline 0.9%). Signaling events in heart tissue were assessed at 24 h subsequent to CLP. Each immunoblot is from a single experiment and is representative of five separate experiments. Densitometric analysis of the bands is expressed as relative optical density (OD) of (**a**) phosphorylated Akt (pSer^473^) corrected for the corresponding total Akt content (Σ Akt); (**b**) phosphorylated eNOS (pSer^113^) corrected for the corresponding total eNOS content (Σ eNOS). All values were corrected for the corresponding Tubulin and normalized using the related sham-operated band. The following groups were studied: sham + vehicle; CLP + vehicle; CLP + Pep2.5. Data are expressed as means ± SD for *n* number of observations. *P < 0.0001 vs. CLP + vehicle (Kruskall-Wallis test with Dunn’s multiple comparisons test).

As activation of Akt and endothelial nitric oxide synthase (eNOS) improve cardiac function during sepsis^22^, we investigated the effects of Pep2.5 on the degree of phosphorylation of Akt on Ser^473^ (Fig. 7a) and eNOS on Ser^133^, respectively (Fig. 7b). When compared with sham animals, mice subjected to CLP showed a significantly decreased phosphorylation of Akt on Ser^473^ (*P* = 0.0036) (Fig. 7a) and eNOS on Ser^133^ (*P* = 0.0072), respectively (Fig. 7b). In contrast, the intravenous administration of Pep2.5 (2.0 μg/h in 0.9% saline, 100 μl/h) significantly increased the phosphorylation of Akt on Ser^473^ (*P* < 0.0001) (Fig. 7a) and eNOS on Ser^133^ (*P* < 0.0001) (Fig. 7b), when compared to sham or CLP-challenged mice without specific treatment.

### Effect of Pep2.5 treatment on SERCA2 expression in cardiomyocytes exposed to serum from septic shock patients

**Figure 8:**
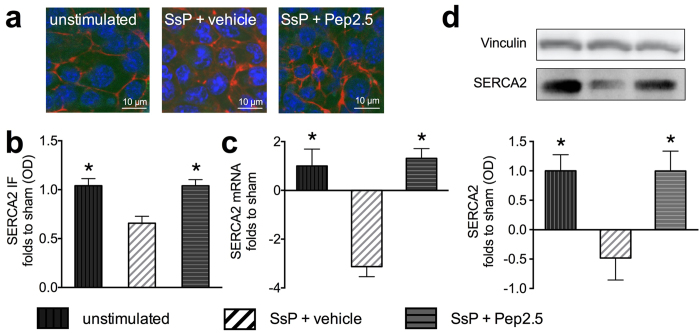
Effect of treatment with Pep2.5 on SERCA2 expression in cardiomyocytes exposed to serum from septic shock patients. Cardiomyocytes (HL-1 cells) were exposed to medium (unstimulated) or serum from septic shock patients (SsP; 5%) in medium and treated with Pep2.5 (20 μg/ml in medium) or vehicle (medium) for 24 h. (**a**) Representative immunofluorescence images of cardiomyocytes stained against sarcoplasmic reticulum Ca2+-ATP-ase (SERCA2) (green), nucleus (blue) and cytoskeletal F-actin (red). (**b**) Quantification of SERCA2 expression in cardiomyocytes was assessed by immunofluorescence and shown as relative optical density (OD); (**c**) Representative immunoblot of SERCA2 (bottom) and vinculin (top). OD of SERCA2 bands corrected for the corresponding vinculin bands and normalized using the related sham band; (**d**) Relative mRNA expression of SERCA2 corrected for S7 expression and normalized to sham. The following groups were studied: unstimulated cells; SsP + vehicle; SsP + Pep2.5. All experiments have been performed in triplicates. Data are expressed as means ± SD for five independent experiments performed in triplicates. *P < 0.0001 vs. SsP + vehicle (Kruskall-Wallis test with Dunn’s multiple comparisons test).

Having shown that the intravenous administration of Pep2.5 significantly attenuates the decrease of SERCA2 mRNA (*P* < 0.0001) and SERCA2 protein expression in the mouse heart, we aimed to confirm these findings in cardiomyocytes exposed to serum from septic shock patients, as a translational approach. When compared to unstimulated cells, cardiomyocytes exposed to human sepsis serum (for 24 h) showed a significant decrease of SERCA2 mRNA (*P* < 0.0001) and SERCA2 protein expression measured by western blot (*P* < 0.0001) and immunofluorescence (*P* = 0.0009) (Fig. 8). The addition of Pep2.5 (20 μg/ml), however, significantly attenuated the decrease of SERCA2 mRNA (*P* < 0.0001) and SERCA2 protein expression measured by western blot (*P* < 0.0001) and immunofluorescence (*P* = 0.0009) in cardiomyocytes exposed to human serum from septic shock patients (Fig. 8).

## Discussion

This study demonstrates for the first time that an infusion of the antimicrobial peptide Pep2.5 (started at onset of CLP-sepsis) preserved systolic and diastolic contractility when measured at 24 h after onset of CLP (Fig. 2). Moreover, Pep2.5 significantly improved the survival time of CLP-challenged mice (Fig. 3). These findings confirm and extend recently published work showing anti-inflammatory effects of Pep2.5 in a murine model of polymicrobial sepsis^12^. In this study, we reported that the infusion of Pep2.5 decreases pro-inflammatory cytokines and CD14 mRNA expression in heart tissue as well as increases the physical appearance and behaviour (activity) in mice subjected to CLP^12^. Notably, CD14 is important in mediating the proinflammatory response induced by LPS in the heart and the presence of CD14 is essential for the development of left ventricular dysfunction in LPS-challenged mice^23^. Mechanistically, Pep2.5 neutralizes microbial immunostimulatory cell wall constituents such as LPS^8^. Through binding to LPS, Pep2.5 changes the aggregate structure of LPS, thereby preventing its binding to the LPS-binding protein and CD14 and subsequently to Toll-like receptor 4 (TLR4). However, the activity of Pep2.5 is not restricted to Gram-negative bacterial infection^8^, as Pep2.5 reduces NF-κB activation in cardiomyocytes exposed to either LPS, lipoproteins or serum from septic shock patients^14^. Today, evidence exists that TLRs on cardiomyocytes initiate a NF-κB dependent inflammation during sepsis, which leads to myocardial contractile dysfunction^24^,^25^, which is associated with a poor outcome^26^. Indeed, in heart tissue of mice subjected to CLP, we observed a significant increase in the nuclear translocation of NF-κB subunit p65 (Fig. 6a) as well as a significantly increased degree of phosphorylation of IKKα/β on Ser^176/180^ (Fig. 6b) and of IκBα on Ser^32/36^ (Fig. 6c), all of which were abolished following treatment with Pep2.5. IκBα masks the nuclear localization signals of NF-κB proteins and sequesters NF-κB as an inactive complex in the cytoplasm, thereby inhibiting NF-κB^27^,^28^. Signal-induced proteolytic degradation of IκBα, which has been phosphorylated by IκB kinases (IKKα/β) liberates NF-κB to translocate to the nucleus and enter the nucleus^28^. Subsequently, NF-κB activates the transcription of a number of genes involved in producing pro-inflammatory cytokines and chemokines known to contribute to septic cardiomyopathy^29^ (see Supplementary Fig. S3). Thus, the cardioprotective effects of Pep2.5 in sepsis are associated with a significant reduction in the activation of the NF-κB pathway, and, hence, with an attenuated cardiac inflammation.

Furthermore, our results demonstrate that treatment with Pep2.5 activates the well-described Akt/eNOS survival pathway. Akt is a member of the phosphoinositide-3-kinase (PI3K) signal transduction enzyme family. When activated (resulting in phosphorylation of Ser^473^) by it’s upstream regulator PI3K, Akt controls inflammatory response, chemotaxis and apoptosis, thus modulating cell survival and growth^30^. In particular, activation of Akt attenuates the cardiac dysfunction caused by sepsis in mice^18^,^31^,^32^. We document here that CLP-challenge results in a significant reduction in phosphorylation of Akt on Ser^473^, when compared to sham animals (Fig. 7a). In contrast, Pep2.5 prevented the decline in Akt-phosphorylation caused by CLP. Most notably, the degree of Akt-phosphorylation in the heart of septic mice treated with Pep2.5 was even higher than that observed in sham-animals (Fig. 7a). Thus, the cardioprotective effects of Pep2.5 in sepsis are associated with the increased phosphorylation of Akt on Ser^473^ resulting in the activation of the Akt survival pathway in the murine heart tissue.

Additionally, strong evidence exists that activation of Akt is crucial for the phosphorylation of eNOS and, hence, activation of eNOS in cardiomyocytes^33^. Indeed, here we show that the preservation of cardiac function in sepsis afforded by Pep2.5 is associated with a significantly increase on Ser^113^ phosphorylation of eNOS and, hence, eNOS activation (Fig. 7b). During sepsis, eNOS activation exhibits beneficial effects, as activated eNOS enhances the formation of NO. Activation of eNOS causes local vasodilation and inhibition of platelets and attenuates cardiac dysfunction in sepsis^18^,^31^,^32^. Thus, our results suggest that the therapeutic effect of Pep2.5 on cardiac function in CLP-challenged mice is (at least in part) attributable to an anti-inflammatory and a cardioprotective effect mediated by a supressed NF-κB activation, subsequent IKK modulation, and notably the activation of the Akt/eNOS survival pathway.

Having discovered that Pep2.5 exhibits its therapeutic potential in anti-inflammatory and cardioprotective effects, we then investigated it’s effect on the intracellular Ca^2+^ transporter SERCA2. The importance of Ca^2+^ handling in cardiomyocytes becomes evident if one considers that the upper mentioned signalling pathways, including NF-κB and Akt/eNOS, can only contribute to cardiac contractility through effects on Ca^2+^ handling, by either affecting the transient rise in cytosolic Ca^2+^ or the myofilament function.

After systolic transient rise in cytosolic Ca^2+^, the cardiomyocytes relax because Ca^2+^ is removed from the cytosol by reuptake into the SR via SERCA2^5^. There is very good evidence that the diastolic Ca^2+^ re-uptake is reduced during endotoxemia^34^, due to the inhibition of activity or expression of SERCA2^5^. Although SERCA2 suppression has been demonstrated in a dose-dependent fashion in mice 4 and 7 h after endotoxemia^21^, our study is the first investigating SERCA2 expression in a murine model of polymicrobial sepsis mimicking the clinical syndrome (Fig. 4). In mice subjected to CLP both SERCA2 mRNA and protein levels were supressed and this was directly associated with a significant impairment in cardiac function (Fig. 5). To date, there is very limited information about the factors and signalling pathways responsible for the SERCA2 dysregulation during septic cardiomyopathy. Recently, Hadri and colleagues demonstrated that eNOS expression is increased in SERCA2-overexpressing human coronary endothelial cells as a result of SERCA2-mediated activation of the eNOS promoter^35^, demonstrating a potential synergetic effect of SERCA2 and eNOS in the prevention of cardiac dysfunction. Moreover, the activation of NF-κB reduces SERCA2 mRNA in cardiomyocytes^36^. Our *in vivo* investigations reporting an association between the expression of SERCA2 and the activation of NF-κB (Fig. 6) and the Akt/eNOS survival pathway (Fig. 7) strengthen these *in vitro* data. However, further studies are needed to investigate the causative signalling events responsible for the dysregulation of SERCA2 during septic cardiomyopathy.

Interestingly, preservation of cardiac function in sepsis by Pep2.5 was associated with prevention of the down-regulation of SERCA2 expression in murine heart tissue (Fig. 5). These findings support our hypothesis that therapeutic approaches, which prevent the down-regulation of SERCA2 may reduce cardiac dysfunction and improve outcome in patients/animals with sepsis.

Having shown that Pep2.5 prevents SERCA2 down-regulation in murine polymicrobial sepsis, we aimed to confirm these findings in cardiomyocytes exposed to serum from septic shock patients, as a translational approach. Here we demonstrate for the first time that the exposure of cardiomyocytes to the serum obtained from patients with septic shock results in a down-regulation in cardiomyocyte SERCA2 expression (Fig. 8), which was abolished by Pep2.5 (Fig. 8). These findings extend earlier work showing anti-inflammatory effects of Pep2.5 in cardiomyocytes exposed to serum from septic shock patients^14^. Thus, it is likely that the beneficial effect of Pep2.5 treatment on SERCA2 expression is causal to the reduced inflammatory response in the cardiomyocytes exposed to serum from septic shock patients. Although the signalling pathways involved in the regulation of SERCA2 expression have yet to be evaluated, our findings support the view that Pep2.5 may reduce the cardiac dysfunction caused by sepsis in patients. Prior to any clinical (phase I) investigations with this peptide, preclinical toxicological studies in two species are warranted to reduce the likelihood of any potential adverse effects in man. In the last years, several studies have investigated the toxicological profile of Pep2.5: Measuring the respiratory rates of Jurkat cells in a chip-based cellular system^37^ and the hemolysis of red blood cell compared to that of melittin from bee venom, cell cytotoxic effects of Pep2.5 have been reported in the concentration range of 30 to 50 μg/ml. Moreover, potential cytotoxic effects of Pep2.5 have been evaluated in human macrophages and red blood cells. Using the Alamar blue test for macrophages and the efflux of haemoglobin for red blood cells, some cytotoxic effects have been reported for concentrations above 30 μg/ml^38^. Furthermore, according to the ICH (international conference on harmonisation) M3 regulation, repeated dose toxicology was performed with Pep2.5 in rats (Aurigon, Muenchen, Germany). The results of this (unpublished) study indicate that the NOAEL (no observed adverse effect level) is > 3 mg/kg per day and the maximum tolerated dose is >20 mg/kg per day. Thus, the doses of Pep2.5 used in this study are below those that triggered adverse effects in previous toxicological studies.

In conclusion, our results show for the first time that the infusion of Pep2.5 attenuates the impaired systolic and diastolic contractility and improves the survival time in polymicrobial sepsis. Preservation of cardiac function in sepsis by Pep2.5 is, at least in part, attributable to an anti-inflammatory and cardioprotective effect mediated by a supressed NF-κB activation and the activation of the Akt/eNOS survival pathways. Most notably, the cardioprotective effects of Pep2.5 are associated with prevention of the downregulation of SERCA2 expression in hearts of mice with sepsis. Thus, we speculate that Pep2.5 may be useful to influence cardiac SERCA2 expression in patients with sepsis, which, in turn, may improve cardiac function and outcome in these patients.

## Additional Information

**How to cite this article**: Martin, L. *et al.* The synthetic antimicrobial peptide 19-2.5 attenuates septic cardiomyopathy and prevents down-regulation of SERCA2 in polymicrobial sepsis. *Sci. Rep.* **6**, 37277; doi: 10.1038/srep37277 (2016).

**Publisher’s note:** Springer Nature remains neutral with regard to jurisdictional claims in published maps and institutional affiliations.

## Supplementary Material

Supplementary Information
