# Supplementary material for: The synthetic antimicrobial peptide 19-2.5 attenuates septic cardiomyopathy and prevents down-regulation of SERCA2 in polymicrobial sepsis
Source: Sci Rep. 2016 Nov 17;6:37277. doi: 10.1038/srep37277 (PMC5112529; doi:10.1038/srep37277)
Supplement: Supplementary Information [file srep37277-s1.pdf]

SUPPLEMENTARY INFORMATION

The synthetic antimicrobial peptide 19-2.5 attenuates septic cardiomyopathy and prevents down-regulation of SERCA2 in polymicrobial sepsis

Lukas Martin, Klemens Horst, Fausto Chiazza, Silvia Oggero, Massimo Collino, Klaus Brandenburg, Frank Hildebrand, Gernot Marx, Christoph Thiemermann, Tobias Schuerholz

METHODS

Human materials

Table S1. Characteristics of serum from septic shock patients used for the exposure to cardiomyocytes. APACHE II: Acute Physiology and Chronic Health Evaluation score II, IL-6: interleukin-6.

Patient no.	Age (years)	Infecting organism	Serum IL-6 level (pg/ml)	APACHE II score	28-day outcome
1	75	E.coli	5206.5	22	alive
2	82	E.coli	702.9	29	dead
3	76	Ent. aerogenes	546.6	10	alive
4	84	Staph. aureus	575.5	30	alive
5	60	Staph. epidermidis	957.1	19	alive
6	63	Strept. anginosus	2156.2	20	dead
7	75	E. coli	15738.7	25	alive
8	81	E. faecalis	15898.7	22	dead
9	73	E. faecium	5153.8	21	dead
10	79	Klebsiella pneumoniae	14698.9	24	alive

Reagents and compounds

Reagents and compounds were purchased from Sigma Aldrich (Poole, Dorset, United Kingdom), unless otherwise stated. Antibodies for immunoblot analysis were purchased from Santa Cruz Biotechnology (Heidelberg, Germany) unless otherwise stated.

Murine sepsis model

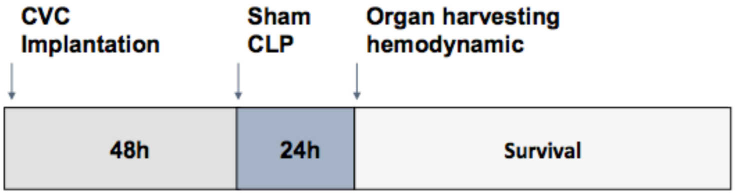

Figure S2. Experimental setup of the murine sepsis model. CVC, central venous catheter; CLP, cecal ligation and puncture

Quantification of cardiac dysfunction

Cardiac function was assessed in mice 24 h subsequent to CLP as described previously[1]. Mice were anaesthetised with 0.5% inhaled isoflurane in oxygen in the same fashion as described before[2,3]. To prevent hypothermia, all animals were kept on a heating pad throughout the surgical procedure. A small incision was made in the anterior neck to expose the right carotid artery. A 1.4-F catheter with pressure and conductance sensors (SPR 839, Millar Instruments, Houston, Texas, USA) was implanted in the right carotid artery and adjusted to obtain optimal flow-volume curves. In the following, the catheter was interfaced with a pressure-volume analogue signal amplifier (Millar Instruments, Houston, Texas, USA) and real-time baseline global

hemodynamic and contractility data were collected using an analogue-to-digital converter (Power Laboratory 4SP, ADInstruments, Castle Hill, Australia) and displayed using the manufacturer's software (Labchart, ADInstruments, Castle Hill, Australia). Mean arterial pressure (MAP), heart rate (HR), stroke volume (SV), left ventricular ejection fraction (LVEF), cardiac output (CO), stroke work (SW), and pressure development during isovolumic contraction (dp/dt_{max}) and relaxation (dp/dt_{min}), were obtained. Next, we evaluated left ventricular contractility under altered preload conditions by transient occlusion of the inferior vena cava (IVCO) with a cotton tip applicator from the opened abdominal cavity[13]. This methodical approach enables the measurement of the left ventricular performance independently from loading conditions[13]. Time-varying maximum elastance (E_{max}) was obtained from the series of pressure-volume relationship regression curves at varying preload levels produced by IVCO. Likewise, preload-recruitable stroke work (PRSW) data were generated with the varying left ventricular end-diastolic volumes. To eliminate parallel conductance, we calibrated with hypertonic saline, as described before[13].

Sampling and laboratory measurements

Blood and hearts were harvested for the quantification of cardiac injury and systemic inflammatory response 24 h subsequent to CLP or sham. Therefore, the animals were anaesthetised as described before[2,3]. Blood and hearts were sampled and snapfrozen in liquid hydrogen. After centrifugation (5000 rpm for 10 min), plasma was separated from cellular blood components. Cytokine profile measurements was performed as described before[3].

RESULTS

Effects of polymicrobial sepsis and treatment with Pep2.5 on plasma cytokines as markers of the inflammatory response in mice. To confirm the recently shown anti-inflammatory effects of Pep2.5[3], we measured plasma cytokines' levels in mice. When compared to the sham animals, mice subjected to CLP demonstrated a significant increase in interleukin-6 (IL-6; $P = 0.0002$), interleukin-10 (IL-10; $P = 0.0249$), monocyte chemoattractant protein-1 (MCP-1; $P < 0.0001$), C-X-C motif ligand 1 (CXCL1; $P < 0.0001$), and interleukin-1β (IL-1β; $P < 0.0001$), indicating a strong proinflammatory response (**Fig. S3**). There were no significant differences in levels of tumor necrosis factor-α (TNF-α; $P = 0.7244$) (**Fig. S3**). The intravenous administration of Pep2.5 (2.0 µg/h in 0.9% saline, 100 µl/h) significantly attenuated this inflammatory response, indicated by significantly lower levels of the plasma cytokines' levels IL-6 ($P = 0.0198$), IL-10 ($P = 0.0054$), MCP-1 ($P = 0.0005$), CXCL1 ($P = 0.0001$), and IL-1β ($P < 0.0001$), respectively (**Fig. S3**). There were no significant differences in the levels of TNF-α ($P = 0.8480$) (**Fig. S3**).

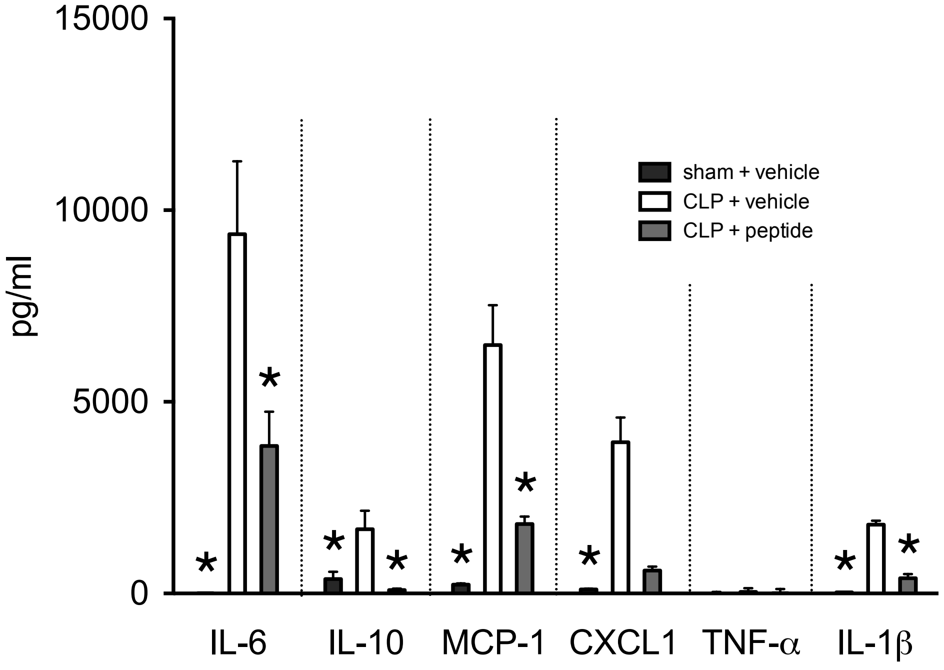

Figure S3: Effect of cecal ligation and puncture (CLP) and treatment with Pep2.5 on plasma cytokines in mice. Plasma levels of interleukine-6 (IL-6), interleukine-10 (IL-10), monocyte chemoattractant protein-1 (MCP-1), C-X-C motif ligand 1 (CXCL1), tumor necrosis factor-α (TNF-α), and interleukine-1β (IL-1β) were assessed 24 h subsequent to sham or CLP in 2-month-old male NMRI-mice. After CLP mice were treated with Pep2.5 (2.0 µg/h in saline 0.9%) or vehicle (100 µl/h saline 0.9%). The following groups were studied: sham + vehicle (n = 8); CLP + vehicle (n = 8); CLP + Pep2.5 (n = 8). Data are expressed as means ± SD for *n* number of observations. *$P < 0.05$ vs. CLP + vehicle (Kruskall-Wallis test with Dunn´s multiple comparisons test).

REFERENCES

1. Pacher, P., Nagayama, T., Mukhopadhyay, P., Sandor B. & Kass, D. A. Measurement of cardiac function using pressure-volume conductance catheter technique in mice and rats. *Nat Protoc* **3,** 1422–1434 (2008).
2. Martin, L. *et al.* The Synthetic Antimicrobial Peptide 19-2.5 Interacts with Heparanase and Heparan Sulfate in Murine and Human Sepsis. *PloS one* **10,** e0143583–13 (2015).
3. Schuerholz, T. *et al.* The anti-inflammatory effect of the synthetic antimicrobial peptide 19-2.5 in a murine sepsis model: a prospective randomized study. *Crit Care* **17,** R3 (2013).
